# Supplementary material for: Whole-Genome Survey Analyses Provide a New Perspective for the Evolutionary Biology of Shimofuri Goby, Tridentiger bifasciatus
Source: Animals (Basel). 2022 Jul 27;12(15):1914. doi: 10.3390/ani12151914 (PMC9367431; doi:10.3390/ani12151914)
Supplement: Supplementary file 1 [file animals-12-01914-s001.zip › animals-1785277-supplementary.pdf]

Table S1. NCBI accession numbers of 13 protein-coding genes of the mitochondrial genomes of 27 species in 9 genera of Gobiidae.

Species	Accession Number
Periophthalmodon schlosseri	NC_030766.1
Acanthogobius stigmothonus	MT258987.1
Acanthogobius hasta	JX186192.1
Boleophthalmus boddarti	NC_023468.1
Boleophthalmus pectinirostris	MN909967.1
Periophthalmus magnuspinnatus	KT357639.1
Periophthalmus modestus	AP019406.1
Periophthalmus novemradiatus	NC_038226.1
Periophthalmus minutus	NC_037073.1
Periophthalmus argentilineatus	NC_029368.1
Tridentiger obscurus	MF663787.1
Tridentiger trigonocephalus	NC_029738.1
Tridentiger kuroiwae	LC653490.1
Tridentiger barbatus	JX536694.1
Lesueurigobius friesii	NC_052760.1
Chaenogobius gulosus	NC_027193.1
Rhinogobius cliffordpopei	KX898434.1
Rhinogobius rubromaculatus	NC_037144.1
Rhinogobius brunneus	NC_028435.1
Rhinogobius leavelli	NC_044964.1
Rhinogobius formosanus	MT363639.1
Rhinogobius duospilus	MH127918.1
Rhinogobius giurinus	KU871066.1
Mugilogobius abei	NC_023353.1
Mugilogobius myxodermus	NC_036070.1
Mugilogobius chulae	NC_026519.1
